# Genome-wide transcriptome analysis of the early developmental stages of *Echinococcus granulosus* protoscoleces reveals extensive alternative splicing events in the spliceosome pathway

**DOI:** 10.1186/s13071-021-05067-9

**Published:** 2021-11-12

**Authors:** Mohammad Ali Mohammadi, Majid Fasihi Harandi, Donald P. McManus, Mehdi Mansouri

**Affiliations:** 1 Student Research Committee, Afzalipour School of Medicine, Kerman University of Medical Sciences, Kerman, Iran; 2 Research Center for Hydatid Disease in Iran, Afzalipour School of Medicine, Kerman University of Medical Sciences, Kerman, Iran; 3 Molecular Parasitology Laboratory, QIMR Berghofer Medical Research Institute, Brisbane, QLD Australia; 4 Department of Agricultural Biotechnology, Faculty of Agriculture, Shahid Bahonar University of Kerman, Kerman, Iran

**Keywords:** Transcriptomics, Echinococcosis, Hydatid disease, Functional analysis, Pepsin, Protoscoleces, Transcription regulation

## Abstract

**Background:**

The complex life cycle of *Echinococcus granulosus* involves numerous environmental conditions within different intermediate and definitive hosts. This requires adaptation at different levels of transcript regulation. Alternative splicing (AS) and the related cellular functions as one of the major fields of post-genomics has been poorly studied in tapeworms. In the present study, we investigated AS events and their potential biological effects in *E. granulosus*.

**Methods:**

Whole transcriptome sequencing data of four groups of protoscoleces were prepared for RNA-seq library construction. Fresh protoscoleces were either used as non-induced controls (NT group) or incubated for 15 min with pepsin (PEP group) and cultivated in a biphasic medium for 12 and 24 h (12 and 24 h groups). The frequency and different types of AS events were identified using rMATS software. Functional annotations and gene ontology of differential AS (DAS) genes were performed using Blast2GO software. AS events were experimentally validated by PCR on the protoscolex cDNAs using specific primers for each gene.

**Results:**

At least one AS event was found in 38.1% of the genes (3904 out of 10,245) in the protoscoleces during early strobilar development. The genes were associated primarily with cellular and metabolic processes and binding and catalytic activity. KEGG pathway analysis of DAS events revealed a number of genes belonging to different components of the spliceosome complex. These genes tended to belong to common SR proteins, U1-related factors, U2-related factors, complex A-specific factors and other splicing-related proteins.

**Conclusions:**

The high number of AS events in the transcriptome regulatory mechanisms indicates the essential rapid molecular processes required by the parasite for adaptation in different environments.

**Graphical Abstract:**

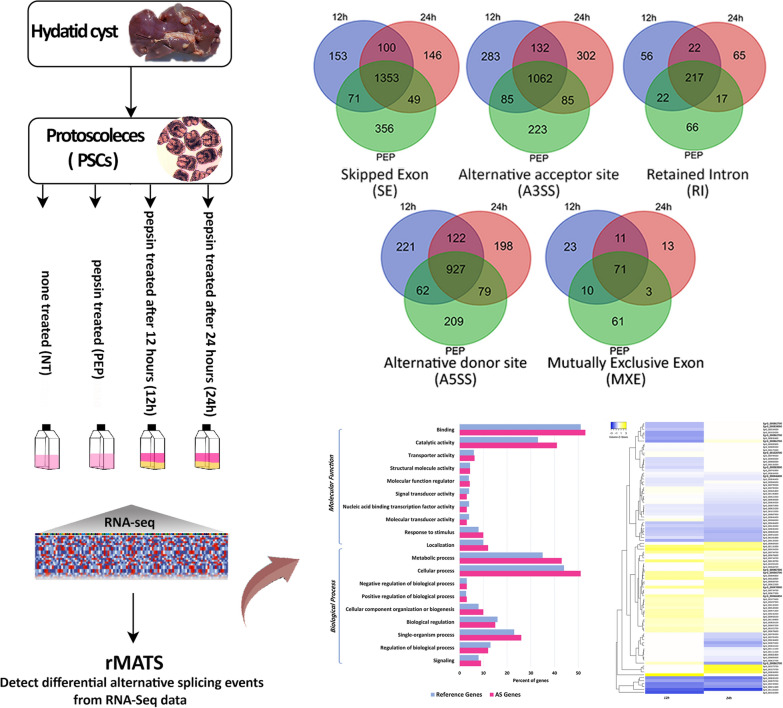

**Supplementary Information:**

The online version contains supplementary material available at 10.1186/s13071-021-05067-9.

## Background

*Echinococcus* species are small intestinal tapeworms of carnivores that cause echinococcosis, one of the most significant neglected zoonotic infections [1]. Cystic and alveolar echinococcosis, caused by *Echinococcus granulosus* and *Echinococcus multilocularis*, respectively, are the two most common forms of echinococcosis. *Echinococcus granulosus* has a two-host life cycle with a large variety of herbivorous mammals as intermediate hosts carrying the larval stage of the helminth, known as hydatid cysts, mainly in the liver and lungs. Different domestic and wild carnivores act as the definitive hosts harboring the adult worms in the intestine. The definitive host acquires the infection through ingestion of hydatid cysts containing viable protoscoleces in the internal organs of the intermediate host [2].

*Echinococcus granulosus* has been considered as a model organism for studying developmental biology and host–parasite interactions in platyhelminths [3, 4]. The study of the *E. granulosus* life cycle becomes more interesting given the protoscoleces’ fascinating ability to develop bidirectionally into the strobilated worm and microcysts in diphasic and monophasic culture media, respectively. Because of the nature of the life cycle, it is essential for the parasite to exploit beneficial strategies to adapt to the different environments in different hosts/organs. Therefore, to ensure maximum morphological/physiological compatibility, the parasite needs to make major changes in the its cellular and molecular processes. However, the basic molecular mechanisms of these strategies require further studies [5].

The role of pepsin in the process of parasite transformation from naive invaginated protoscoleces to evaginated protoscoleces has been documented [6]. The time required for evagination in the definitive host varies between 6 and 72 h, and it is critical for the parasite to be quickly positioned and attached to the surface of the gut to avoid being swept out [7]. Therefore, it is believed that major molecular and cellular events occur during the early differentiation of the parasite. However, the precise nature of these events is not fully understood [8]. Understanding the molecular mechanisms underlying parasite differentiation may provide a window through which to gain insight into the development of *E. granulosus* in the definitive hosts.

The ability of *E. granulosus* to respond quickly to environmental changes in the host body requires changes in different levels of gene expression regulation and protein synthesis. One of the essential characteristics of eukaryotic gene expression is a post-transcriptional process called alternative splicing (AS), which is governed by the spliceosome pathway [9]. The spliceosome is a multi-megadalton ribonucleoprotein complex with highly dynamic conformation and composition, which undoubtedly has critical roles in diverse cellular processes and the accuracy and flexibility of splicing processes [10–12]. AS is one of the main sources of proteome diversity and alternatively spliced isoforms in higher eukaryotes. Stage-specific changes in splicing and subsequent changes in gene expression and hypervariability of the parasite proteins are potential confounding factors in vaccine and drug development [13, 14]. For instance, albendazole, the most important antiparasitic drug in the treatment of echinococcosis, prevents correct assembly and function of the parasite cytoskeleton. Among several β-tubulin gene isoforms in *Echinococcus* species, the highest levels of gene expression are identified as *tub-1*, *tub-2* and *tub-3* [15]. Although *tub-1 and tub-3* are highly expressed in the larval stages of the parasite, and this is the main reason for the general sensitivity of the parasite to albendazole, the most abundantly expressed isoform in all life cycle stages of *E. multilocularis* is *tub-2*, which encodes a potentially resistant phenotype to benzimidazoles. On the other hand, disturbances in AS could be detrimental to parasite survival and proliferation, making it a worthy potential drug target to pursue. As an example, some protein kinase inhibitors generate aberrant AS processes that protect against Duchenne muscular dystrophy or Alzheimer’s disease [16, 17].

It has been shown that coordinated splicing networks regulate different levels of tissue/organ development and essential physiological functions in different developmental processes [18]. The spliceosome is assembled from five small nuclear RNAs (snRNAs) and approximately 80 associated protein factors. This combination of RNA–protein complexes, called small nuclear ribonucleoproteins (snRNPs), provides the main spliceosome (U1, U2, U4, U5 and U6) [11, 19, 20]. Spliceosomes catalyze ligation of the flanking exons after removal of introns that enable one gene to encode more than one mature messenger RNA (mRNA). AS is a critical flexible ability with a vital role in the translation of genetic codes from DNA to proteins by increasing the coding capacity of genomes [21]. Primary modes of AS have been categorized into five main groups: skipped exon (SE), mutually exclusive exons (MXE), alternative donor site (A5SS), alternative acceptor site (A3SS) and retained intron (RI) [22].

The identification and quantification of active genome regions (transcripts) and final products of expressed transcripts (proteins) are of fundamental importance to elucidate biological functions [23]. Next-generation sequencing technologies, as a revolutionary tool, have dramatically improved sequencing time, cost, throughput and accuracy. Notably, the annotation of genes in the reference genome of many organisms is still far from complete, and many novel genes/transcripts remain to be identified [24, 25]. Accordingly, next-generation RNA sequencing (RNA-seq) has emerged as a revolutionary tool for an in-depth, genome-wide view of the transcriptome. RNA-seq has considerable advantages for the detection of novel transcripts, allele-specific expression and AS in comparison with other methods such as microarray techniques [26].

Recently, genomic and transcriptomic resources have been combined with high-throughput sequencing methods for investigating gene expression during different developmental stages of platyhelminths including *Schistosoma mansoni* and the cestode *E. multilocularis* as model systems [27]. To some extent, the mechanisms of pre-mRNA splicing have been characterized in certain metazoan organisms; however, the significance of spliceosomal proteins in developmental biology of the flatworms is still poorly understood [20, 28, 29].

The structure of the *E. multilocularis* gene encoding U1 small nuclear RNA, which is involved in RNA splicing, was previously described and compared with other *Echinococcus* species [30]. Liu et al. recently described AS events in the genus *Echinococcus*. It was found that AS occurs in more than one-third of *E. granulosus* and *E. multilocularis* genes of significant importance in numerous pathways, particularly those related to metabolism, signal transduction and gene regulation [31]. However, the nature and extent of AS events in the early developmental phases of *E. granulosus* have not been explored. The process of evagination and early development of the protoscoleces in the dog intestine, particularly the first 24 h after ingestion, is a critical phase of *E. granulosus* development towards strobilation. Because of the high frequency and potential versatility of AS in many fundamental biological processes, such as cell differentiation and specification, in this study we investigated AS events in the early strobilar stages of *E. granulosus* development and their potential impacts on the spliceosome pathways.

## Methods

### Parasite materials

Protoscoleces of *E. granulosus* were collected from a hepatic hydatid cyst of a naturally infected sheep slaughtered at a local municipal abattoir. The specimens were transferred to the Research Center for Hydatid Disease in Iran, and the viability of the protoscoleces was determined by the eosin dye-exclusion test and their motility examined using light microscopy. Protoscoleces were washed three times with phosphate-buffered saline (PBS) and immediately processed for further molecular characterization. DNA was extracted using the High Pure PCR Template Preparation Kit (Roche, Germany) according to the manufacturer’s instructions. DNA was used for *E. granulosus* genotype identification using polymerase chain reaction (PCR) sequencing of cytochrome c oxidase as described by Bowles et al. [32]. The *E. granulosus* sensu stricto G1 genotype was used in the experiments. Protoscoleces from the same cyst/same genotype (G1) were used in all in vitro culture treatments in order to minimize the differences among the samples.

### RNA sequencing, data preprocessing and mapping

Whole-transcriptome sequencing data used in this study (NCBI SRA database: SRP131874) were generated and the following procedure was used, as described in detail elsewhere [33]. The protoscoleces were cultured as described by Debarba et al. 2015 [34]. Briefly, after washing the protoscoleces in PBS, they were used either as non-induced controls (NT group) or for strobilation induction, and were treated as follows. The protoscoleces were incubated for 15 min with pepsin (2 mg/ml), pH 2.0 (PEP group), washed with PBS and cultivated in a biphasic medium containing taurocholate for 12 h (12 h group) or 24 h (24 h group). Transcriptome libraries for each group were constructed using the TruSeq Stranded mRNA LT Sample Prep Kit (Illumina) and sequenced in a MiSeq Sequencing System (Illumina) with 2 × 75 base-pair (bp) paired-end reads.

FastQC v0.11.8 [35] was used for RNA-seq dataset quality checking. Individual sequencing read files (FASTQ) were trimmed and filtered using Trimmomatic software v0.36 [36] with paired-end parameters as follows: LEADING:10, TRAILING:10, SLIDINGWINDOW:30:20, MINLEN:30. Finally, quality-filtered reads in FASTQ format were trimmed to 76 nucleotides using the trimFastq Python script provided by rMATS software v3.2.5 [37]. The high-quality trimmed reads were mapped to the published genome of *E. granulosus* (PRJEB121, Tsai et al., 2013) and its annotation (version 2014-05) in WormBase ParaSite WBPS13 (WS269) using the STAR aligner [38].

### Differential alternative splicing analysis

The five types of differential alternative splicing (DAS) events, including SE, A5SS, A3SS, MXE and RI, were identified using rMATS. Annotated and novel AS events for each group of parasites were extracted from an aligned BAM file and high-quality transcript reference GTF file. The AS events were identified based on inclusive and exclusive reads that spanned the splicing junction or occurred in the alternative exon, intron target inclusion count (IC) or skipping count (SC). Expressed AS events and the associated AS genes, including replicates, were revealed when a total inclusion junction count (IJC) of ≥ 1 and total skipping junction count (SJC) ≥ 1 were found in all samples [37]. rMATS evaluated DAS events in each group of protoscoleces when the difference in the exon or intron inclusion level of AS events occurring between two samples exceeded a stringent threshold (FDR ≤ 0.05). Significant events of junction count with reads on target were calculated by comparing PEP, 12 and 24 h with NT groups.

### Functional annotation and Gene Ontology (GO) enrichment analysis

Functional annotations and gene ontology of DAS genes were performed using BLAST searches against the NCBI non-redundant protein and InterPro scan databases using Blast2GO software [39]. The AgriGO v2.0 online analysis toolkit was used to identify overrepresented GO terms within the AS genes of *E. granulosus* [40]. The hypergeometric statistical test was applied to the false discovery rate (FDR) under dependency using the Hochberg (FDR) multiple-testing adjustment method to retrieve significant GO terms in comparison to the whole genome background (*P* < 0.05). Comparisons of the transcripts with the Kyoto Encyclopedia of Genes and Genomes (KEGG) database was performed for genes with DAS events using the bidirectional best hit (BBH) method in the KEGG Automatic Annotation Server (KAAS) against available helminth species (flatworms, Cestoda, and the nematode organism model *C. elegans*) for ortholog assignment and pathway mapping [41].

### Reverse transcriptase polymerase chain reaction validation of AS events

Triple batches of the protoscoleces (for each of the NT, PEP, 12, and 24 h groups) were used with the same experimental conditions to evaluate AS events in the early stages of *E. granulosus* development. For each of the batches, triplicate reactions of reverse transcriptase PCR (RT-PCR) were carried out for AS event validation. Total RNA from each of the protoscolex batches was individually extracted using TRIzol reagent (YTA Co., Iran), according to the manufacturer's instructions. Any contamination by genomic DNA was eliminated with DNase I (Thermo Scientific, USA) treatment in all RNA extractions, and complementary DNA (cDNA) was synthesized from 100 ng of total RNA using a cDNA synthesis kit (YT4500, YTA, Iran).

AS events were experimentally validated by performing PCR on the protoscolex cDNAs with a specific primer for each gene, which was designed to amplify both isoforms of each splice variant in a single reaction. Among significant DAS events in all four groups of *E. granulosus*, five homologous genes with the highest inclusion-level differences (IncLevelDifference > 0.5) were randomly selected for RT-PCR validation. Primers flanking predicted splicing events were designed using PrimerSeq software [42] (Additional file 1: Table S1). Conventional PCR was carried out with a total of 20 µl of mixed reagents based on the following conditions: initial denaturation at 95 °C for 5 min, followed by 30 thermal cycles of 95 °C for 30 s, 59 °C for 60 s and 72 °C for 90 s, and a final extension of 72 °C for 10 min. Finally, 5 µl of the PCR product was electrophorized and visualized on 1.5% agarose gel. Using the Genome Browser tool in WormBase ParaSite, additional exploration was carried out against the *E. granulosus* genome to compare various representative transcripts (splice variants) of the selected genes.

## Results

### AS events in the early developmental stages of *E. granulosus*

**Fig. 1:**
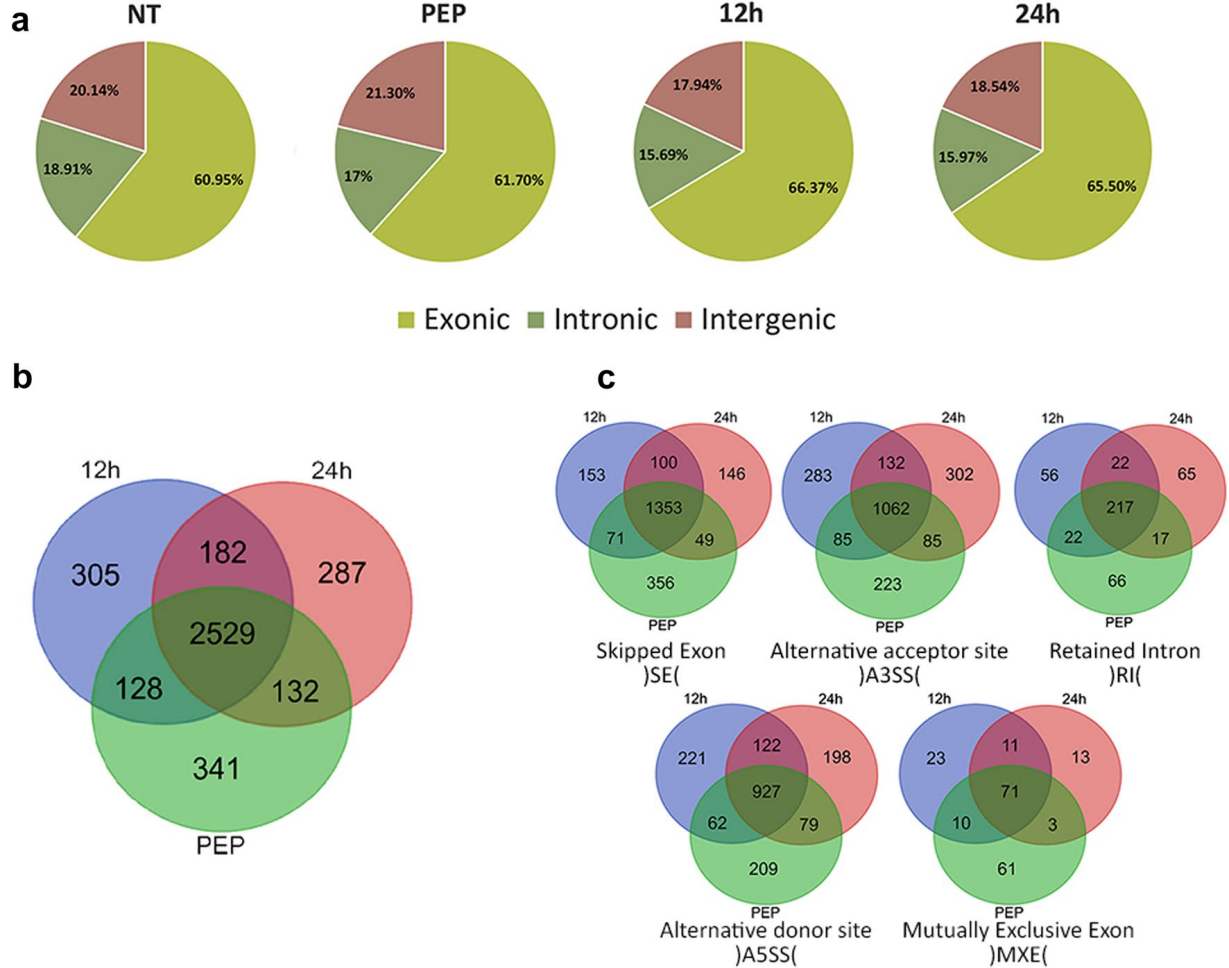
**a** Distributions of sequence read mapping of the *E. granulosus* genome, indicating similar patterns of exon and intron percentages. **b** Total distribution of 3904 genes with alternative splicing events in pepsin-treated groups. **c** Frequency distribution of five different modes of alternative splicing events in the early developmental stages of *E. granulosus*. *NT* Non-treated group, *PEP* pepsin-treated protoscoleces, *12 h* 12 h after pepsin treatment, *24 h* 24 h after pepsin treatment

To investigate the AS events on a genome-wide scale, RNA-seq experiments were performed on *E. granulosus* protoscoleces examined at different time points, 0, 12 and 24 h, after initial pepsin treatment. The comprehensive profiling of the AS landscape at these conditions was conducted by using the same RNA‐Seq data with the overall high-quality reads mean with Q30 quality score 98.3% (Table1). In total, 30,966,414 high-quality reads were obtained, of which 48.4–54.7% were uniquely mapped to the *E. granulosus* genome. The sequence reads were classified into three clusters with exon frequency of 60.95–66.37%, introns of 15.69–18.91% and intergenic 17.94–21.3% (Fig. 1a, Additional file 1: Table S2). A total of 23,558 AS events were predicted, corresponding to 3904 genes out of 10,245 *E. granulosus* coding genes (38.1%) (Fig. 1b and 1c and Additional file 3: Datasheet S1). Among the five types of AS events, SE was the most frequent AS type (9226 events) in all treated groups, followed by A3SS and A5SS with 6841 and 5652 events, respectively. RI and MXE, with 1276 and 563 events, respectively, were the least common splicing forms in the early strobilar stages of *E. granulosus*. Comparison of the three treatment groups revealed that SE was the most highly shared AS event (1353 genes) followed by A3SS (1062 genes) and A5SS (927 genes) (Table 2).

**Table 1:** Summary of RNA-seq data obtained from pepsin-treated *E. granulosus* protoscoleces at 0, 12 and 24 h post-treatment

Samples	Q30^a^	High-quality reads	Total read length (bp)	Mapped reads with STAR (%)
NT	0.984	6,994,764	528,313,589	49.1
PEP	0.983	7,348,062	554,809,442	48.4
12 h	0.985	8,308,408	627,289,019	54.7
24 h	0.982	8,315,180	627,939,696	53.4

*NT *non-treated, *PEP* Initial pepsin treatment, 12 h 12 h pepsin treatment, 24 h 24 h pepsin treatment

^a^Q30: Phred Quality Score; the probability of incorrect base call: 1 in 1000

**Table 2:** Identification of stage-specific differential alternative splicing events in early strobilar stages of *E. granulosus* as provided by rMATS

Sample type	PEP			12 h			24 h			Total events	
Event type	NumEvents^a^	SigEvents^b^	(NT: PEP) ^c^	NumEvents	SigEvents	(NT:12 h)	NumEvents	SigEvents	(NT:24 h)	NumEvents	SigEvents
SE	3386	0	(0:0)	2937	53	(14:39)	2903	47	(15:32)	9226	100
MXE	235	0	(0:0)	173	1	(0:1)	155	2	(0:2)	563	3
A5SS	1834	0	(0:0)	1910	2	(1:1)	1908	3	(1:2)	5652	5
A3SS	2147	0	(0:0)	2322	0	(0:0)	2372	1	(1:0)	6841	1
RI	428	1	(1:0)	428	14	(8:6)	420	13	(9:4)	1276	28
Total events	8030	1	–	7770	70	–	7758	66	–	23,558	137

*SE* skipped exon, *MXE* mutually exclusive exon, *A5SS* alternative 5′ splice site, *A3SS* alternative 3′ splice site, *RI* retained intron

^a^Total number of events detected using both junction counts and read on target

^b^Number of significant events detected using both junction counts and read on target

^c^The numbers in parentheses indicate the number of significant events with a higher inclusion level

### DAS events in the early developmental stages of *E. granulosus*

Altogether, 137 significant DAS events were found across 68 genes in the pepsin-treated groups (Table2, Additional file 1: Table S3). The total number of AS events was similar among all three groups; however, the PEP group had only one significant DAS event in comparison to the NT group. The total number of DAS events was calculated as 70 and 66 for the 12 and 24 h groups, respectively.

The results showed that only a single DAS event, oxalate/formate antiporter (EgrG_000661800), was observed between NT and PEP samples. In the early strobilar stages of *E. granulosus,* SE was the most common significant DAS type in all the treated groups (particularly in 12 and 24 h groups), with 100 events, followed by RI with 28 events (Additional file 1: Table S3).

### Validation of the predicted AS events

**Fig. 2:**
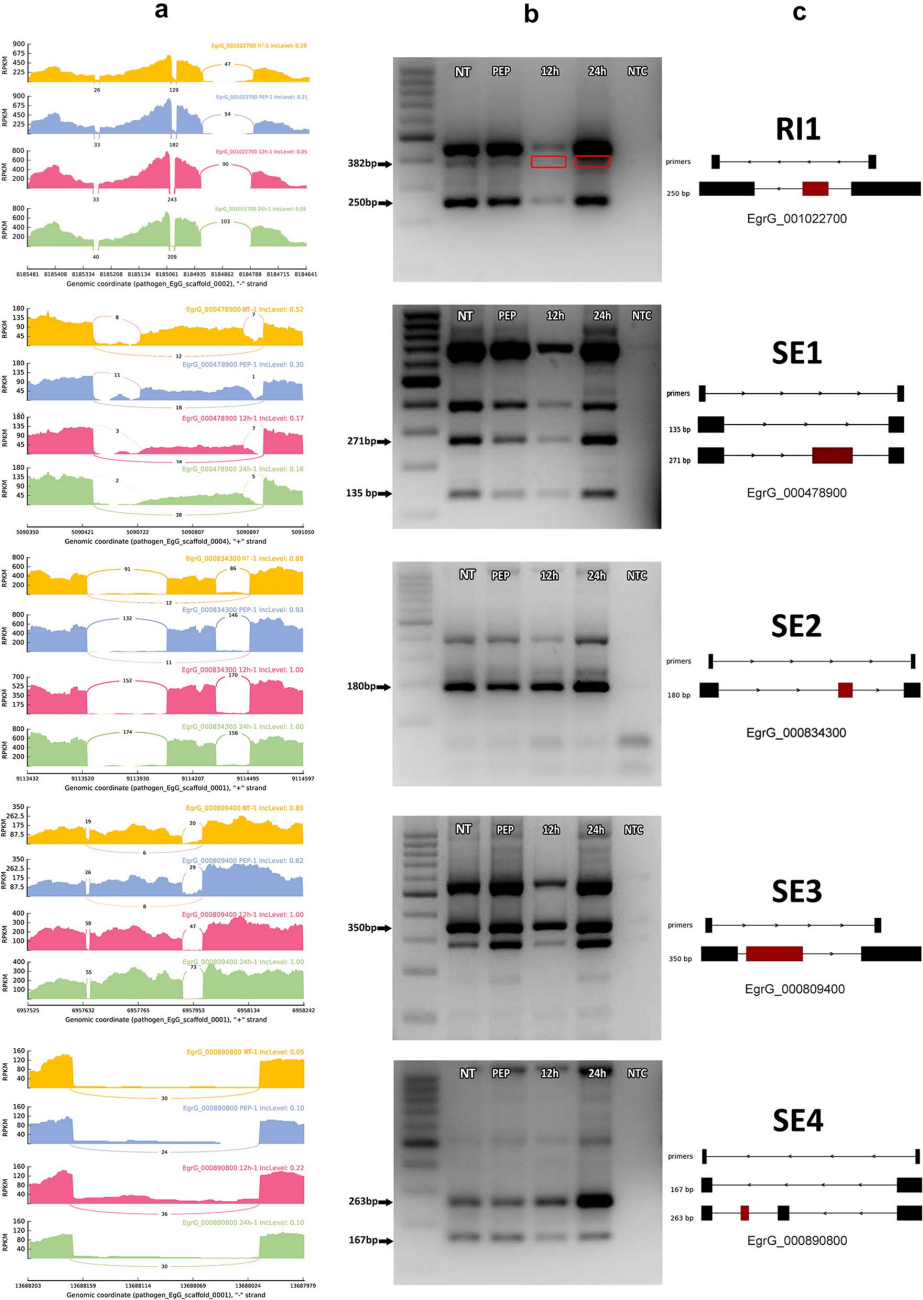
Reverse transcriptase PCR validation of skipped exon (SE) and retained intron (RI) events in the early developmental stages of *E. granulosus* protoscoleces at different time points after induction of strobilar development. **a** Sashimi plot depicting the region tested for the presence of alternative splicing isoforms. Non-induced control protoscoleces (NT group), pepsin-treated protoscoleces (PEP group), and protoscoleces cultivated in biphasic medium for 12 and 24 h (12 and 24 h groups). The exons with alternative splicing events were described as expected base-pair size of the transcript with and without exon splicing along with average inclusion level (IncLevel) based on DNA band densitometry. **b** Agarose gel electrophoresis of transcript isoform amplification corresponding to each treatment group. Red frames show the missing bands indicating retained intron AS event. *NTC* non-template control for PCR amplifications. **c** A subset of retained intron event (RI1) and skipping exon events (SE1, SE2, SE3 and SE4) in the protoscoleces were confirmed by RT-PCR. Schematic frames show predicted AS events in exons and introns. In panel c, the exon is highlighted in red. For a detailed view of the full gene and splicing isoforms, see Additional file 2: Figures S1–S6

Reverse transcriptase PCR (RT-PCR) was carried out for one gene with an RI event: EgrG_001022700 (AN1 type zinc finger protein 5); and four genes with SE events: EgrG_000478900 (survival of motor neuron splicing factor), EgrG_000834300 (tetraspanin), EgrG_000809400 (Cdc42; Ras gtpase) and EgrG_000890800 (FERM domain-containing protein 3). The reliability of the bioinformatics prediction of AS events was validated by amplification of both splice isoforms (Fig. 2). Some other extra bands were observed in the PCR products, suggesting additional AS events from the corresponding genes. There were some differences in band size due to the use of new predicted isoforms and because of the criteria defined in PrimerSeq for detecting/introducing the amplification region (Additional file 2: Figures S2–S6).

The exon inclusion level (a popular statistical index which indicates the percent spliced in) showed a clear correlation between the NT and PEP groups versus 12 and 24 h treatments. In this regard, the exon inclusion level (IncLevel) for the RI in EgrG_001022700 was 19% (for NT) and 21% (for PEP), which was greater than 12 and 24 h with 9 and 5% IncLevel, respectively (Fig. 2).

### GO enrichment and pathway analysis

**Fig. 3:**
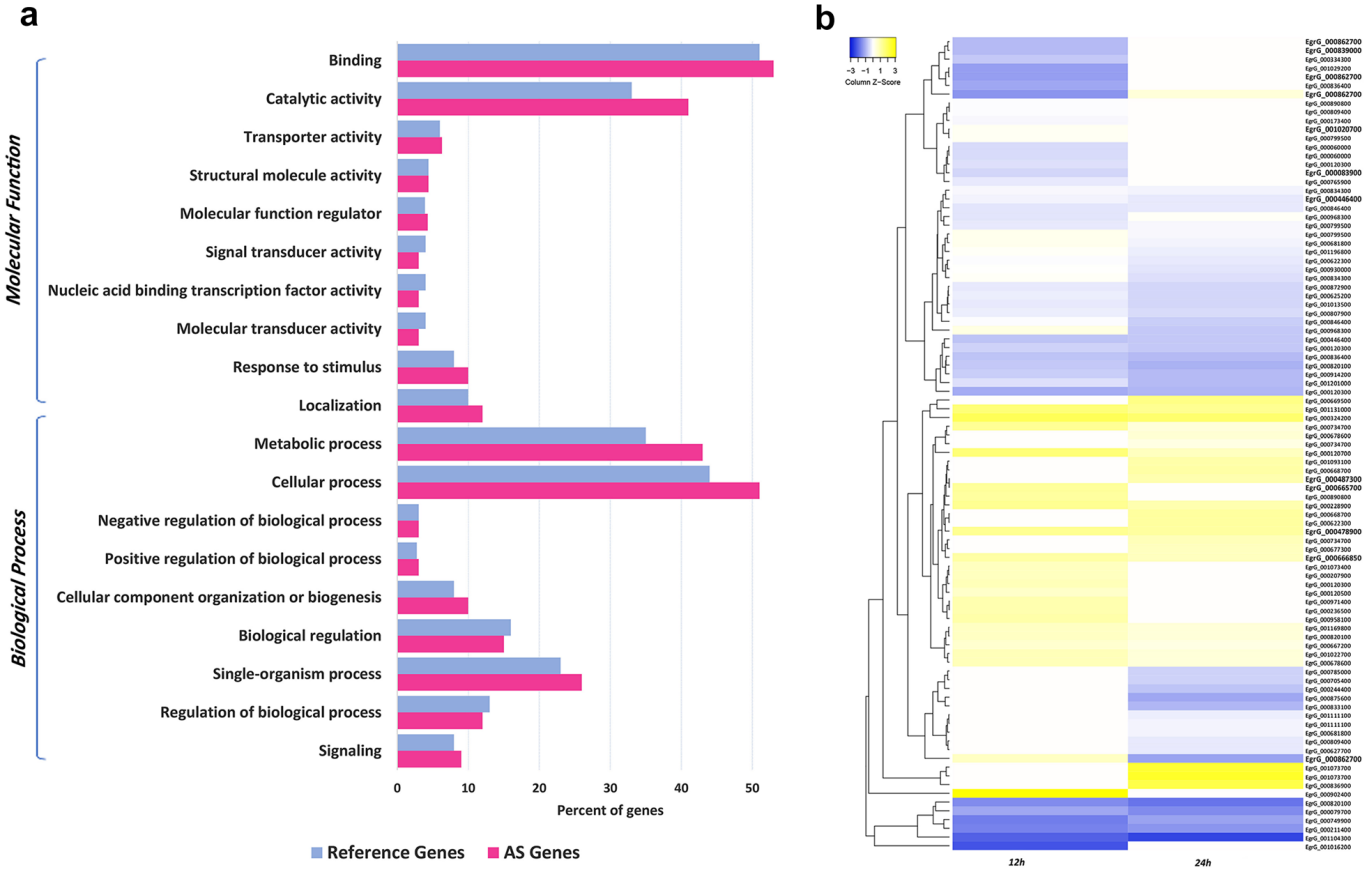
**a** Functional GO enrichment analysis of alternative splicing events in early developmental stages of *E. granulosus* protoscoleces. **b** Changes in the transcript isoforms of the genes with DAS events in pepsin-treated protoscoleces after 12 and 24 h compared to the non-treated group. Clustering of differential alternative splicing events data is made after scaling inclusion-level differences (Z-score transformation). Genes with differential alternative splicing corresponding to different components of spliceosome complex are indicated in bold. The figure is available in the supplementary file in its original size (Additional file 4: Figure S2)

Functional enrichment analysis identified several enriched GO terms in biological process and molecular function (Fig. 3a, Additional file 5: Datasheet S2 and Additional file 6: Figure S7). The top six highly significant enriched GO terms of AS genes were represented in catalytic activity and ligase activity as molecular function, and organic substance metabolic process, primary metabolic process and cellular process as biological processes (Table 3). The catalytic activity and ligase activity GO terms have considerable relation with Ribosome biogenesis in eukaryotes pathway with 13 genes with at least one AS event (Additional file 1: Table S4).

**Table 3:** Top six highly significant enriched GO terms of AS genes in the early developmental stages of *E. granulosus* protoscoleces

Ontology	Description	Number in the input list	Number in BG/Ref	*P*-value	FDR
Molecular function	Catalytic activity	1140	2238	3.5 × 10^−10^	1.1 × 10^−7^
Biological process	Metabolic process	1192	2457	9.7 × 10^−7^	5.3 × 10^−4^
Biological process	Organic substance metabolic process	1062	2166	1.5 × 10^−6^	5.3 × 10^−4^
Biological process	Primary metabolic process	1025	2095	3.7 × 10^−6^	8.7 × 10^−4^
Biological process	Cellular process	1399	2982	1.7 × 10^−5^	3.0 × 10^−3^
Molecular function	Ligase activity	103	142	2.5 × 10^−5^	4.1 × 10^−3^

*FDR* false discovery rate

### DAS events related to spliceosome complex

Significant changes in the splicing pattern were observed in the early strobilar stages of *E. granulosus*, showing the critical functionality of AS. A much more curious finding in KEGG pathway analysis revealed a number of genes belonging to the different components of the spliceosome complex undergoing AS. Pathway mapping analysis and BRITE hierarchies showed nine coding genes from the spliceosome pathway with at least one AS event (Table 4). *Echinococcus* genes EgrG_000478900 (SMNDC1) and EgrG_000666850 (U2AF1) are part of U2-related factors, and EgrG_000862700 (TCERG1) is part of U1-related components of the spliceosome complex. EgrG_000446400 (SFRS7) is part of the common components of the spliceosome. Also, three genes, EgrG_000839000 (RBM5_10), EgrG_000665700 (SF1) and EgrG_001020700 (TLS), from the complex A-specific factors and two genes of other splicing-related proteins, EgrG_000083900 (HNRNPF_H) and EgrG_000487300 (YTHDC1), were among the genes with DAS events. Interestingly, EgrG_000862700 (TCERG1) has been found to influence the life span pathway of some nematodes, called the longevity-regulating pathway. The high rate of total DAS events in the spliceosome (9 out of 68 genes) was supported by BRITE hierarchies and KEGG Orthology analysis (Additional file 1: Table S5). Changes in the transcript isoforms of genes with DAS events after 12 and 24 h are demonstrated in Fig. 3b. Clustering of DAS events data was conducted after scaling inclusion-level differences (Z-score transformation) (Fig. 3b).

**Table 4:** Genes with differential alternative splicing corresponding to different components of spliceosome complex based on KEGG pathway analysis

Stable gene ID^a^	KEGG Orthology	Gene name	Definition
EgrG_000478900	K12839	SMNDC1	Survival of motor neuron-related splicing factor 30
EgrG_000666850	K12836	U2AF1	Splicing factor U2AF 35 kDa subunit
EgrG_000446400	K12896	SFRS7	Splicing factor, arginine/serine-rich 7
EgrG_000839000	K13094	RBM5_10	RNA-binding protein 5/10
EgrG_000665700	K13095	SF1	Splicing factor 1
EgrG_001020700	K13098	TLS	FUS RNA-binding protein
EgrG_000083900	K12898	HNRNPF_H	Heterogeneous nuclear ribonucleoprotein F/H
EgrG_000487300	K20100	YTHDC1	YTH domain-containing protein 1
EgrG_000862700	K12824	TCERG1^a^	CA150 transcription elongation regulator 1

All genes correspond to spliceosome pathway ko03040

^a^*Echinococcus granulosus* BioProject PRJEB121

^b^TCERG1 corresponds to both spliceosome pathway ko03040 and longevity-regulating pathway–worm ko04212

## Discussion

In the present study we investigated AS events in the early strobilar development of *E. granulosus*. AS and the related cellular functions are a major field of post-genomic research that has been explored in an increasing number of parasite species largely due to the technical and bioinformatics improvements over the past decade [43–45]. The process of evagination of the protoscoleces in the dog intestine is a critical phase of *E. granulosus* development towards strobilation. The majority of protoscoleces evaginate within 6 h after ingestion; however, the process can take up to 72 h to complete. [8]. In the early hours after evagination it is essential for the protoscoleces to exploit beneficial strategies to adapt rapidly and flexibly to the new environmental conditions, such as changes in temperature, osmotic pressure and pH [8, 46]. Different regulatory mechanisms of gene expression such as dynamic changes in microRNA expression in controlling the miRNA–mRNA complex are believed to be involved in the response to these changes [47]. Nevertheless, the precise nature of the physiological and molecular events in the new environment is not fully understood [3, 6, 8].

We found AS events in 38.1% of the genes in *E. granulosus* protoscoleces. This is in accord with the frequency of AS events in other species of platyhelminths. Liu et al. found AS events in 33–36% of the genes in the protoscoleces of *E. granulosus* and *E. multilocularis* [31]. This figure was 35% for adult *Schistosoma japonicum* [48]. In the model organisms *Drosophila melanogaster* and *C. elegans*, AS events occurred in 31% and 25% of the genes, respectively [49, 50]. Obviously, the number of AS events in *E. granulosus* protoscoleces obtained in our study was higher than that of *C. elegans* (25%), but much less than the expected AS events in humans (greater than 90%) [10, 51]. This is an interesting finding; despite the small genome size of *E. granulosus*, the total number of exon/introns (on average 6.8 exons per gene in 10,245 genes) is comparable to that of *C. elegans* (6.4 exons per gene) (Table 5). Although AS is a predictor of developmental complexity, the number of genes undergoing AS in *E. granulosus* still seems to be higher than that in other worms [18, 45]. This may indicate the existence of active regulatory mechanisms in early strobilar development of the parasite adapting to the new environment. It can be inferred that a high exon/intron density is related to the organism's complexity and comparable to the 1.3 billion years of continuous life evolution [52]. More in-depth studies are needed to explain this high level of AS genes in *E. granulosus* metacestodes. In parasitic nematodes, 11.68–31% of the genes were found to be involved in AS [53]. It should be noted that few studies have investigated AS events in different life stages of helminths. However, according to some studies on nematodes and trematodes, it is believed that the larval stages undergo higher rates of AS events compared to the adult stages [48, 53, 54].

**Table 5:** Comparison of the exon density in relation to the genome size and transcripts number in different species of helminths

Organism	Genome Size	Coding genes	Gene transcripts	Number of exons	Exon density	Genes with AS event (%)	BioProject	Reference
*Echinococcus granulosus*	114,538,160	10,245	10,273	69,673	6.8	38.1%	PRJEB121	WormBase ParaSite, Present study
*Echinococcus granulosus*	110,837,706	11,319	11,319	75,264	6.64	33.31%	PRJNA182977	WormBase ParaSite [31]
*Echinococcus multilocularis*	114,963,242	10,663	10,663	72,069	6.76	35.75%	PRJEB122	WormBase ParaSite [31]
*Taenia multiceps*	240,610,560	12,890	12,892	84,541	6.55	nd	PRJNA307624	WormBase ParaSite
*Taenia solium*	122,393,951	12,467	12,467	69,707	5.59	nd	PRJNA170813	WormBase ParaSite
*Taenia saginata*	169,104,283	13,161	13,161	82,095	6.23	nd	PRJNA71493	WormBase ParaSite
*Schistosoma japonicum*	402,743,189	12,738	12,738	67,109	5.26	42.14%	PRJEA34885	WormBase ParaSite [48, 54]
*Schistosoma mansoni*	409,579,008	10,144	14,528	91,980	9.06	> 50%	PRJEA36577	WormBase ParaSite [66]
*Ascaris lumbricoides*	316,975,410	23,604	23,604	141,154	5.98	nd	PRJEB4950	WormBase ParaSite
*Trichuris suis*	71,056,402	14,261	14,261	80,919	5.67	nd	PRJNA208416	WormBase ParaSite
*Trichuris trichiura*	75,496,394	9650	9650	55,156	5.71	nd	PRJEB535	WormBase ParaSite
*Caenorhabditis elegans*	100,286,401	20,184	61,541	147,847	6.4^a^	25%	PRJNA13758	[49]

^*a*^Based on WormBook: The Online Review of  *C. elegans* Biology. (https://www.ncbi.nlm.nih.gov/books/NBK19662/)

Debarba et al. found high correlation coefficients in the two groups of PBS and PEP samples and 12 and 24 h groups, which was highly similar to AS event patterns in our study. Moreover, 75 newly synthesized proteins were reported in the first 24 h of the strobilation process [34]. These differential proteins were mainly involved in metabolic, regulatory and signaling processes, which confirms the essential quick response to environmental changes. In this respect, the previous study on the transcriptomic analysis of the early strobilar development of *E. granulosus* showed several differentially expressed genes following pepsin treatment [33].

In this study, 3904 genes with at least one AS event were predicted. In this regard, 68 unique genes were found with significant DAS events among all four groups of *E. granulosus*. Agarose gel electrophoresis visualized bands in RT-PCR products corresponding to the predicted splice isoforms. Extra bands were found in the gels, implying the additional AS events in the corresponding reference genome. rMATS can detect novel splice sites and exons in annotated transcripts, as well as any novel combinations of annotated and unannotated splice sites that can predict and amplify additional bands [55]. However, our estimate of AS events in *Echinococcus* protoscoleces could be varied due to the effect of the AS process according to the stages, species, tissue type and environmental conditions of the parasite. A lower percentage of AS events were obtained from *S. japonicum* in the adult stages [48] compared to the rate of AS in the schistosomula and adult stages together [54]. As we performed the experiments in vitro, an even higher AS percentage would be expected in an in vivo setting because of the complexity of the parasite life cycle in both intermediate and definitive hosts [31].

Among the five main modes of AS, we found that SE was the most common significant AS type, followed by RI, in all treated groups of early strobilar development of *E. granulosus*, which was confirmed by specific primer sets amplifying five random gene isoforms with significant AS events. These results are comparable to the previous genome-wide transcriptome analyses of AS events in *Echinococcus* species, in which intron retention but not exon skipping was reported as the predominant type of AS [31]. According to the nature of AS events, this discrepancy may be because we examined AS events at different time points of the early stages of the parasite development, which has already been reported in other helminth species [48, 54].

In this study, we performed gene ontology analysis to summarize and explore the functional categories of genes with AS events. The results indicated that the proportions of enriched GO categories of all AS events among the four groups were categorized as biological process and molecular function. In this regard, catalytic activity (GO:0003824) and metabolic process (GO:0008152) were the top significant enriched GO terms of AS genes in the early developmental stages of *E. granulosus* protoscoleces. This is in line with Liu et al., who reported biological process, molecular function and cellular components, with the most relevant AS events corresponding to the cellular process, metabolic process, and binding and catalytic activity [31]. Interestingly, a recent study on the early developmental stages of *E. granulosus* protoscoleces shows that miRNAs and genes are likely associated with nervous system and carbohydrate metabolic processes necessary to prepare the parasite for bidirectional development [47].

KEGG pathway enrichment analysis showed that genes with DAS events were enriched in multiple metabolic pathways. The findings of KEGG pathway analysis of 68 genes with DAS events became more interesting when we found an unexpected number of genes involved in the spliceosome pathway. These genes tended to belong to common SR proteins (SFRS7), U1-related factors (TCERG1), U2-related factors (U2AF1, SMNDC1), complex A-specific factors (RBM5_10, SF1, TLS) and other splicing-related proteins (SMNDC1, HNRNPF_H, YTHDC1).

The role of transcription elongation regulator 1 (TCERG1) in the splicing pathway has been described previously [21]. TCERG1, also known as CA150, is a nuclear protein that regulates transcriptional elongation by regulating RNA polymerase II elongation and pre-mRNA splicing related to splicing factor SF1. The different expression ratios among TCERG1 isoforms between male and female *S. mansoni* was reported earlier [56]. It has been suggested that these differences may play a critical role in the sex evolution of *Schistosoma* species. Interestingly, a transcription elongation regulator gene was found to be upregulated in the encystation process of *E. granulosus* protoscoleces [57].

Based on KEGG Orthology, TCERG1 is involved in the longevity-regulating pathway in worms, and it was shown in *Caenorhabditis elegans* that the *tcer-1* gene encodes the homolog of the human transcription elongation factor TCERG1 [58]. During the past decade, the aging process with its related metabolic pathways has been clearly described in *C. elegans* [59]. Because of the simple biology of *C. elegans*, this organism could prove to be a useful tool for studying the life span in worms, with over 70 characterized genes related to longevity [53, 59]. Although losing germ cells is typically required to extend life span, the overexpression of TCER-1 in worms with normal germ cells is still sufficient to increase *C. elegans* life span. Remarkably, TCER-1 is not essential for DAF-16/FOXO to extend the life span in helminths by reducing insulin/IGF-1 signaling as a central regulator of life span [58], which is a vital signaling pathway in both *E. multilocularis* and *E. granulosus* [5, 60]. The time taken for worms to achieve patency in dogs is estimated at 6 to 8 weeks, but under equilibrium conditions, the life span for an adult *E. granulosus* is estimated at between 1 and 3.5 years from the moment the protoscoleces are eaten by the dog until the adult worms are expelled [8, 61]. Because of the nature of the cyst developmental process in the host body, the study and estimation of the life span of the larval stage is complex. Since regular dog-dosing programs and removal of worms before they mature is one of the key measures in cystic echinococcosis control programs, further studies on longevity-regulating pathways could provide new opportunities for disease control and prevention.

The nature of the spliceosome complex makes splicing a complicated dynamic process that assembles various mRNA by folding exon and intron regions from primary mRNAs. AS can modulate gene expression in multiple ways due to the exon density in the genome of *E. granulosus*. Interestingly, a high AS event rate in *E. granulosus* can be explained by the parasite's fascinating capability for bidirectional development. Protoscoleces can develop into either adult worms in the dog intestine or abdominal cystic stages through secondary infection in the intermediate host [5, 8]. With the limited number of genes in *E. granulosus*, the high rate of AS events offers the parasite a biological advantage to develop this adaptation.

A number of genes with significant AS events in pre-RNA processing are related to catalytic and ligase activity, and this is supported by GO enrichment analysis of RNA-seq data of early developmental stages of *E. granulosus* protoscoleces. In this regard, U3 small nucleolar RNA (snoRNA)-associated proteins (UTP11, UTP13, UTP14, and UTP21) were the most frequent genes with AS events. Recent evidence indicates that snoRNAs play an essential role in the regulation of gene expression; for example, U3 snoRNA plays a critical role in processing small subunit (SSU) rRNA [62, 63]. The presence of this number of genes with AS events in the regulatory mechanisms of gene expression conforms to the rapid adaptation processes of the parasite to different environmental conditions at the molecular level.

Stage-specific variations in splicing and subsequent changes in gene expression and hypervariability of the parasite proteins should be considered in developing vaccines and therapeutic targets [14, 15]. In this regard, transcript isoforms may vary with regard to the number of situations such as parasite life stage, various genotypes and host–parasite interactions [15, 64]. This remains elusive despite decades of research on vaccine development against metazoan parasites.

It should be noted that AS primary modes describe fundamental splicing mechanisms, but AS processes may be much more complicated [22]. We used pepsin as an effective player in the protoscoleces evagination in an in vitro system; however, in vivo investigations of the role of other environmental factors in natural definitive hosts are needed. Regarding the remarkable biological, physiological and epidemiological differences among *E. granulosus* genotypes, there are very likely different key gene isoforms with different levels of expression in the genotypes, resulting in variations in the helminth developmental processes. However, to gain comprehensive insight into the host–parasite adaptation in metazoan parasites, more in-depth studies should be conducted using deep-level RNA-seq datasets and advanced bioinformatics for predicting AS events followed by supporting experimental validations including gene knockdown/knockout and small-molecule inhibition of spliceosomes [65].

## Conclusion

Our understanding of AS events in *E. granulosus* is poor, and very few studies have been conducted on this topic. This study investigated AS events in different strobilar stages of *E. granulosus* in the first 24 h of protoscolex development. In the early hours of evagination, it is essential for the protoscoleces to exploit efficient strategies to flexibly adapt to the changes in the new environment. Despite the limited number of genes in the parasite genome, a higher rate of AS events, associated primarily with cellular and metabolic processes and binding and catalytic activity, was documented in early strobilar development of *E. granulosus*. This may reflect the unexpected number of AS events in the genes involved in RNA biogenesis and the spliceosome pathway. Further studies are needed on the molecular mechanisms involved in the early development of the parasite from the very beginning of parasite entry and its subsequent attachment to the definitive hosts. This will improve our knowledge regarding the probable pathogenic mechanism of echinococcosis in definitive hosts.

## Supplementary Information

**Additional file 1: Table S1**. List of primers used for reverse transcription PCR validation to amplify both isoforms of each splice variant in a single reaction. Primers flanking predicted splicing events were designed using PrimerSeq software. **Table S2**. Proportions of the three types of sequence reads mapping to the genomes of early developmental stages of *Echinococcus granulosus* protoscoleces. **Table S3**. Identified genes with differential alternative splicing events in the early developmental stages of *Echinococcus granulosus* protoscoleces. **Table S4**. List of genes with differential alternative splicing events in the early developmental stages of *Echinococcus granulosus* protoscoleces relating to the catalytic activity and ligase activity in GO analysis of RNA-seq data. **Table S5**. BRITE hierarchy of *Echinococcus granulosus* gene IDs with differential alternative splicing events in the early developmental stages of *E. granulosus* protoscoleces.

**Additional file2: Figure S1**. High-resolution version of Fig. 2; reverse transcription PCR validation of skipped exon (SE) and retained intron (RI) events in the early developmental stages of *E. granulosus* protoscoleces at different time points after induction to strobilar development. **Figures S2–S6.** A comparison of the amplification sites for each selected AS event of different isoforms

**Additional file 3: Datasheet S1**. *Echinococcus granulosus* genes with primary modes of alternative splicing events and differential AS events, predicted by rMATS. *Total SE* total skipped exon (SE) events in the early phases of *E. granulosus* development identified by rMATS, *Total RI* total retained intron (RI) events in the early phases of *E. granulosus* development identified by rMATS, *Total MXE* total mutually exclusive exon (MXE) events in the early phases of *E. granulosus* development identified by rMATS, *Total A3SS* total alternative acceptor site (A3SS) events in the early phases of *E. granulosus* development identified by rMATS, *Total A5SS* total alternative donor site (A5SS) events in the early phases of *E. granulosus* development identified by rMATS.

**Additional file 4: Figure S2**. High-resolution heatmap plot of changes in the transcript isoforms of the genes with differential AS events in 12 h and 24 h groups compared to the non-treated group.

**Additional file 5: Datasheet S2**. Gene ontology analysis of *E. granulosus* genome with AS events in the early phases of development.

**Additional file 6: Figure S7**. High-resolution diagram of gene ontology analysis using online analysis toolkit, AgriGO

## Data Availability

The datasets analyzed during the current study are publicly available on GenBank under the accession number SRP131874. All generated or analyzed datasets are available from the corresponding author on reasonable request.

## Abbreviations

AS: Alternative splicing
DAS: Differential alternative splicing
snRNA: Small nuclear RNA
snRNPs: Small nuclear ribonucleoproteins
SE: Skipped exon
MXE: Mutually exclusive exons
A5SS: Alternative 5′ splice site
A3SS: Alternative 3′ splice site
RI: Retained intron
NT: Non-treated group
PEP: Pepsin-treated group
12 h: Pepsin-treated group after 12 h
24 h: Pepsin-treated group after 24 h
TCERG1: Transcription elongation regulator 1
