# Supplementary material for: Genome-wide transcriptome analysis of the early developmental stages of Echinococcus granulosus protoscoleces reveals extensive alternative splicing events in the spliceosome pathway
Source: Parasit Vectors. 2021 Nov 12;14:574. doi: 10.1186/s13071-021-05067-9 (PMC8587495; doi:10.1186/s13071-021-05067-9)
Supplement: Supplementary file 2 — Additional file2: Figure S1. High-resolution version of Fig. 2; reverse transcription PCR validation of skipped exon (SE) and retained intron (RI) events in the early developmental stages of E. granulosus protoscoleces at different time points after induction to strobilar development. Figures S2–S6. A comparison of the amplification sites for each selected AS event of different isoforms [file 13071_2021_5067_MOESM2_ESM.pdf]

Fig S1.

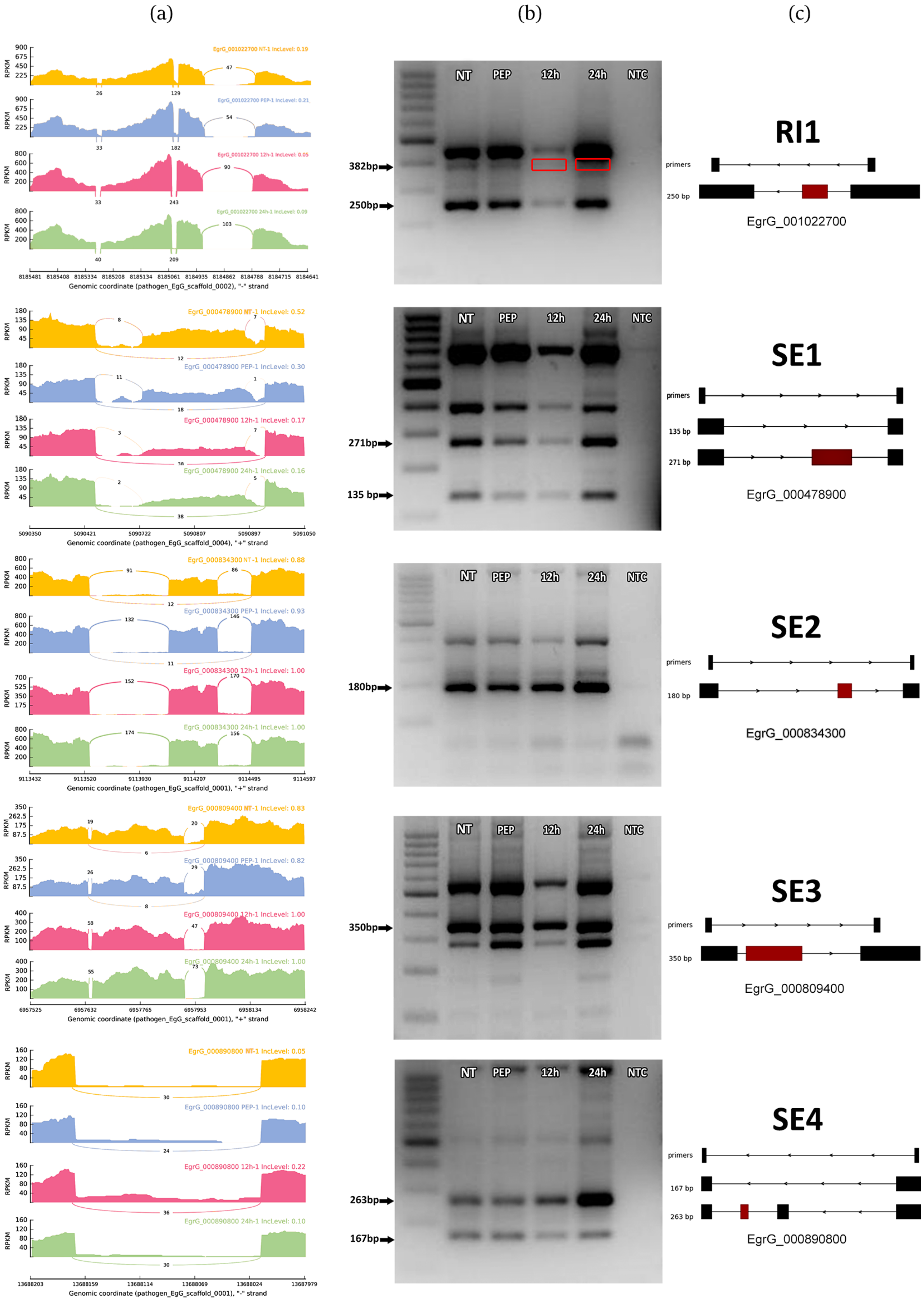

(a)
(b)
(c)
EgrG_001022700 NT-1 IncLevel: 0.19
EgrG_001022700 PEP-1 IncLevel: 0.21
EgrG_001022700 12h-1 IncLevel: 0.05
EgrG_001022700 24h-1 IncLevel: 0.09
Genomic coordinate (pathogen_EgG_scaffold_0002), "-" strand
EgrG_000478900 NT-1 IncLevel: 0.52
EgrG_000478900 PEP-1 IncLevel: 0.30
EgrG_000478900 12h-1 IncLevel: 0.17
EgrG_000478900 24h-1 IncLevel: 0.16
Genomic coordinate (pathogen_EgG_scaffold_0004), "+" strand
EgrG_000834300 NT-1 IncLevel: 0.88
EgrG_000834300 PEP-1 IncLevel: 0.93
EgrG_000834300 12h-1 IncLevel: 1.00
EgrG_000834300 24h-1 IncLevel: 1.00
Genomic coordinate (pathogen_EgG_scaffold_0001), "+" strand
EgrG_000809400 NT-1 IncLevel: 0.83
EgrG_000809400 PEP-1 IncLevel: 0.82
EgrG_000809400 12h-1 IncLevel: 1.00
EgrG_000809400 24h-1 IncLevel: 1.00
Genomic coordinate (pathogen_EgG_scaffold_0001), "+" strand
EgrG_000890800 NT-1 IncLevel: 0.05
EgrG_000890800 PEP-1 IncLevel: 0.10
EgrG_000890800 12h-1 IncLevel: 0.22
EgrG_000890800 24h-1 IncLevel: 0.10
Genomic coordinate (pathogen_EgG_scaffold_0001), "-" strand
RPKM
NT
PEP
12h
24h
NTC
382bp
250bp
271bp
135 bp
180bp
350bp
263bp
167bp
RI1
primers
250 bp
EgrG_001022700
SE1
135 bp
271 bp
EgrG_000478900
SE2
180 bp
EgrG_000834300
SE3
350 bp
EgrG_000809400
SE4
167 bp
263 bp
EgrG_000890800

Fig S2. EgrG_001022700 (RI1)

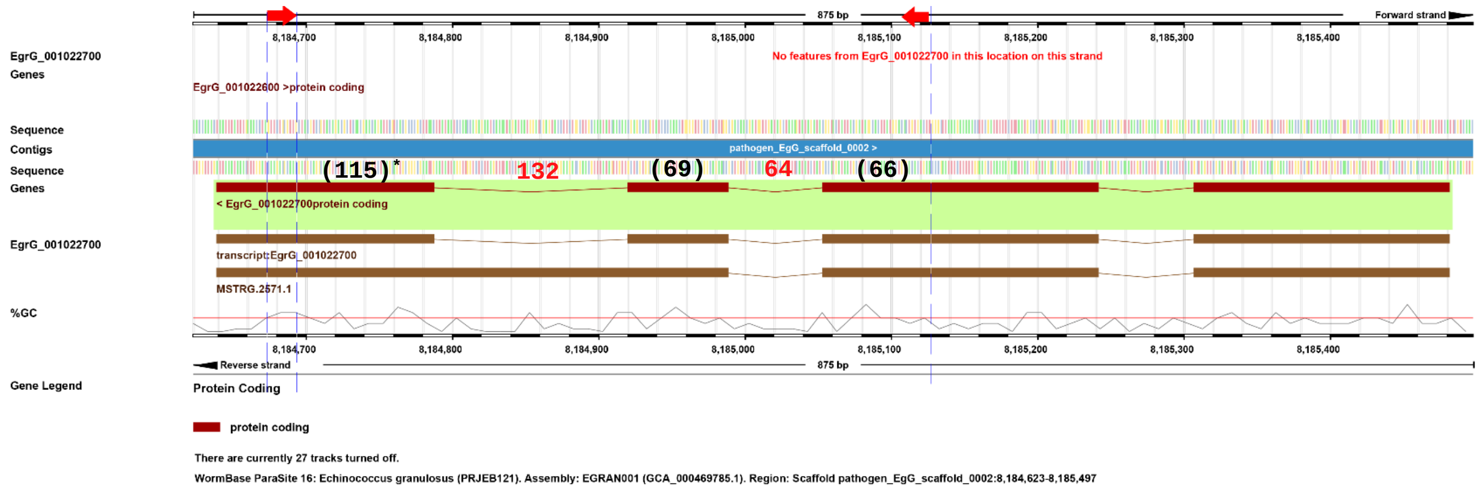

WormBase ParaSite GTF: (E4-E3-E2-E1)

Expected amplification region; partial E4+E3+partial E2= **250 bp**

Novel GTF: (new E3-E2-E1); targeted Exon (-pathogen_EgG_scaffold_0002:8184919-8184988)

Expected amplification region; partial new E3+partial E2=**382 bp**

* In the amplification region, the length of the exons is shown in **(black)**, whereas the length of the introns is shown in **red**.

Fig S3. EgrG_000478900 (SE1)

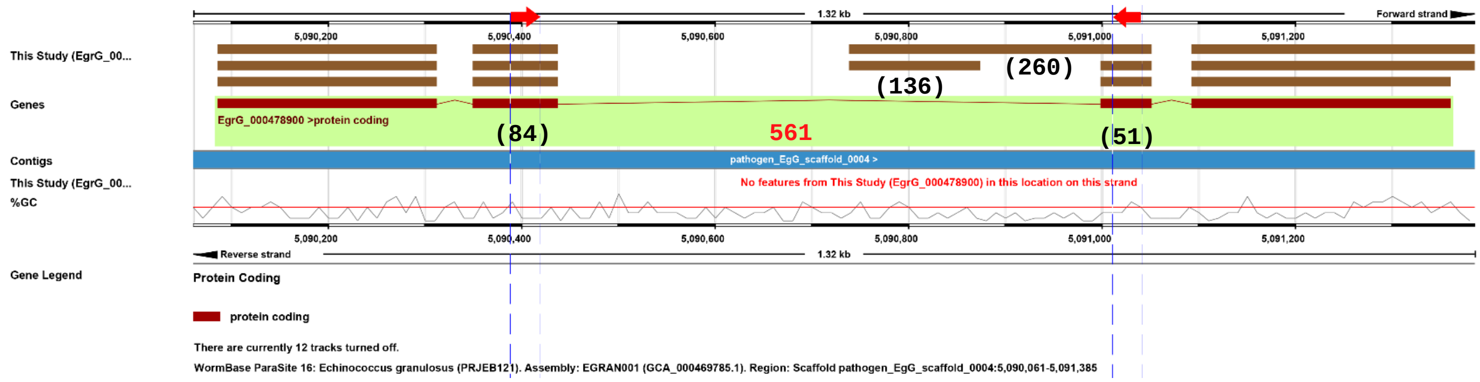

WormBase ParaSite GTF: (E1-E2-E3-E4)

Expected amplification region; partial E2+partial E3= **135 bp**

Novel GTF: (E1-E2-newE3-E4-E5); targeted Exon (+pathogen_EgG_scaffold_0004:5090738-5090874)

Expected amplification region; partial E2+newE3+partial E4=**271 bp**

It should be noted that, this gene has another isoform which is predicted by rMATS"

Novel GTF: (E1-E2-**new longer E3**-E4)

Expected amplification region; partial E2+new longer E3+partial E4=**395 bp**

Fig S4. EgrG_000834300 (SE2)

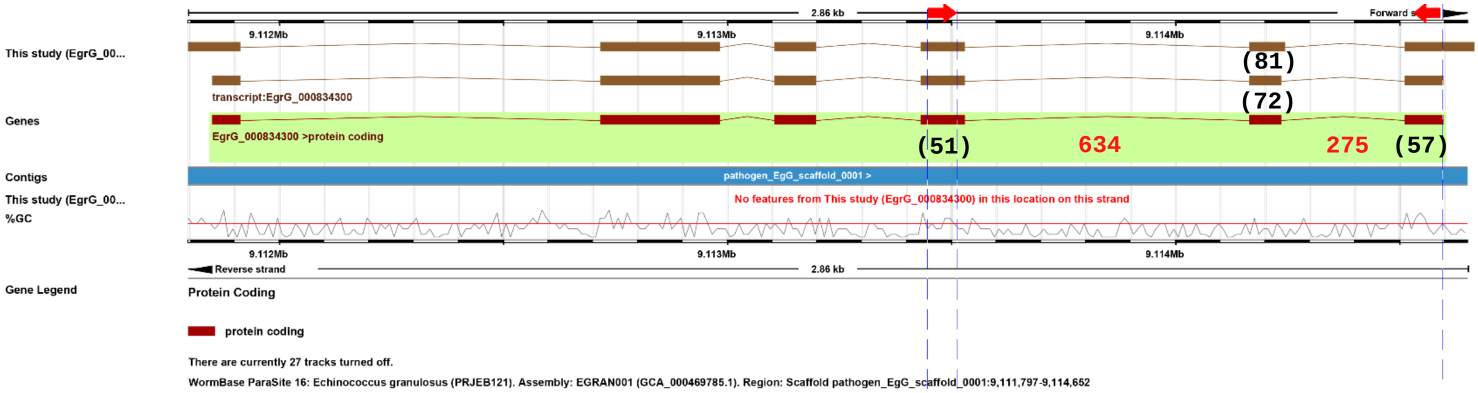

WormBase ParaSite GTF: (E1-E2-E3-E4-E5-E6)

Expected amplification region; partial E4+E5+partial E6= **180 bp**

Novel GTF: (E1-E2-E3-E4-new longer E5-E6); targeted Exon (+pathogen_EgG_scaffold_0001:9114164-9114236)

Expected amplification region; partial E4+new longer E5+partial E6=**189 bp**

Fig S5. EgrG_000809400 (SE3)

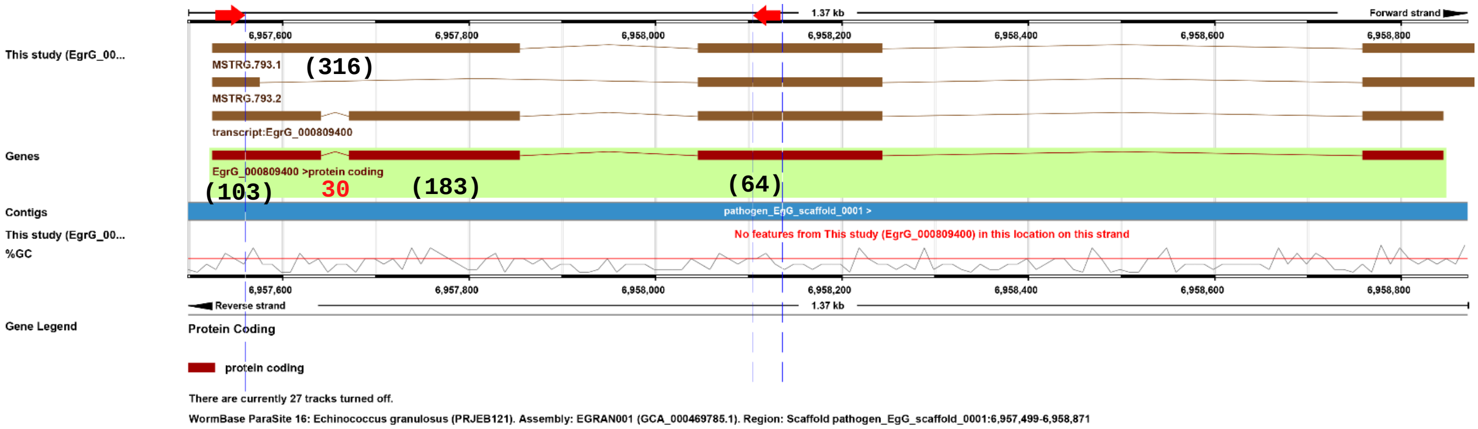

WormBase ParaSite GTF: (E1-E2-E3-E4)

Expected amplification region; partial E1+E2+partial E3= **350 bp**

Novel GTF: (new E1-E2-E3); targeted Exon (+pathogen_EgG_scaffold_0001:6957671-6957854)

Expected amplification region; partial new E1+partial E2=**380 bp**

Fig S6. EgrG_000890800 (SE4)

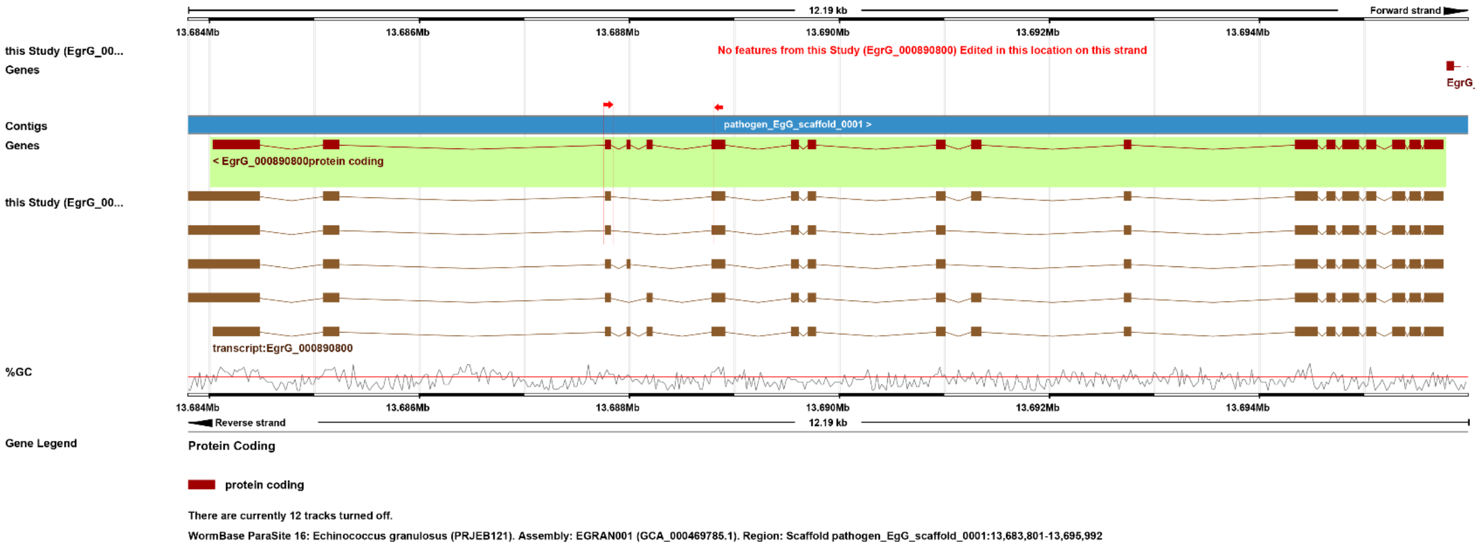

The EgrG_000890800 is long gene with 18 exons (base on Wormbase ParaSite GTF). So, we focused on the targeted region.

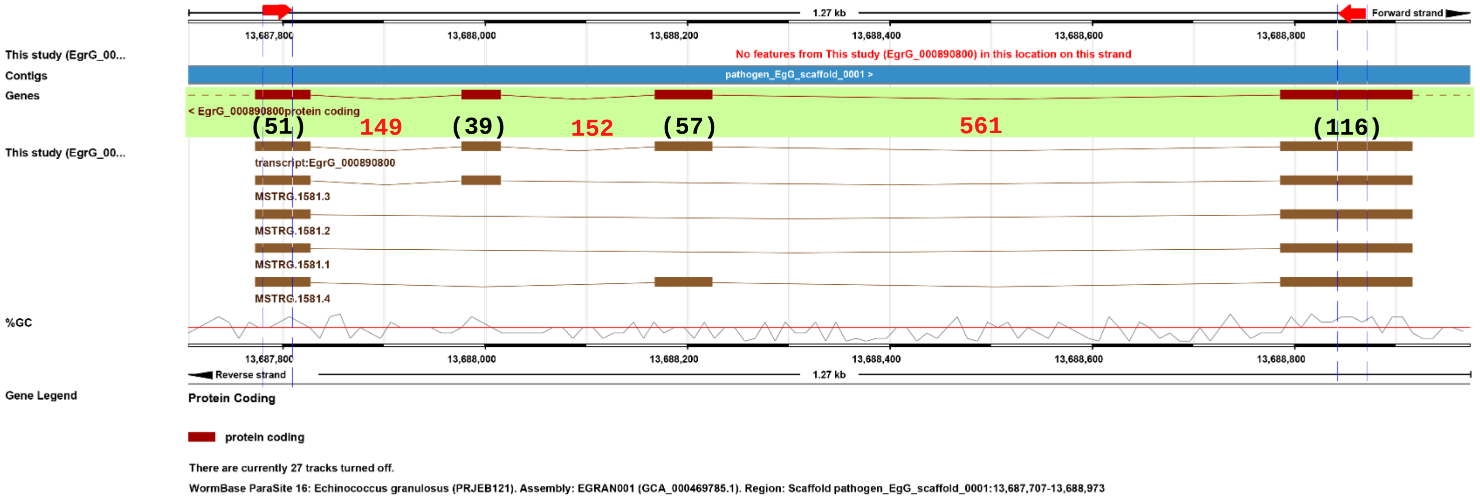

WormBase ParaSite GTF: (E16-E15-E14-E13)

Expected amplification region; partial E16+E15+E14+partial E13= **263 bp**

Novel GTF: (Change in exon number based on AS type); targeted Exons (-pathogen_EgG_scaffold_0001:13687976-13688015) and (-pathogen_EgG_scaffold_0001: 13688167-13688203)

Expected amplification region; partial E16+partial E13=**167 bp**

Novel GTF: (E4-E2-E1); targeted Exon (-pathogen_EgG_scaffold_0001:13687976-13688015)

Expected amplification region; partial E16+E15+partial E13=**206 bp**

Novel GTF: (E4- E3-E1); targeted Exon (-pathogen_EgG_scaffold_0001: 13688167-13688203)

Expected amplification region; partial E16+E14+partial E13=**224 bp**
