# Supplementary figures and images for: Genome-wide transcriptome analysis of the early developmental stages of Echinococcus granulosus protoscoleces reveals extensive alternative splicing events in the spliceosome pathway
Source: Parasit Vectors. 2021 Nov 12;14:574. doi: 10.1186/s13071-021-05067-9 (PMC8587495; doi:10.1186/s13071-021-05067-9)

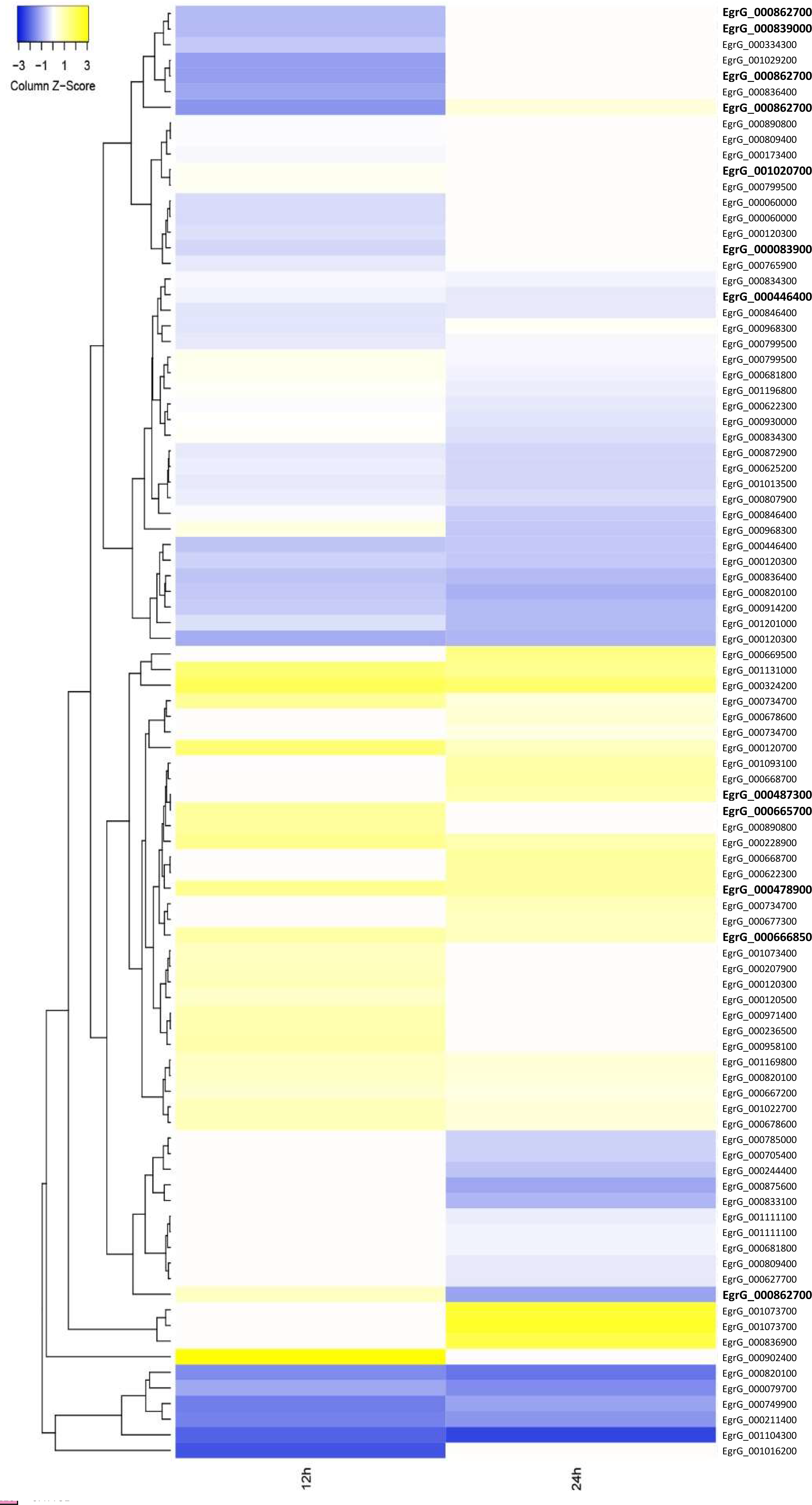

Supplement: Supplementary file 4 — Additional file 4: Figure S2. High-resolution heatmap plot of changes in the transcript isoforms of the genes with differential AS events in 12 h and 24 h groups compared to the non-treated group. [file 13071_2021_5067_MOESM4_ESM.tif]

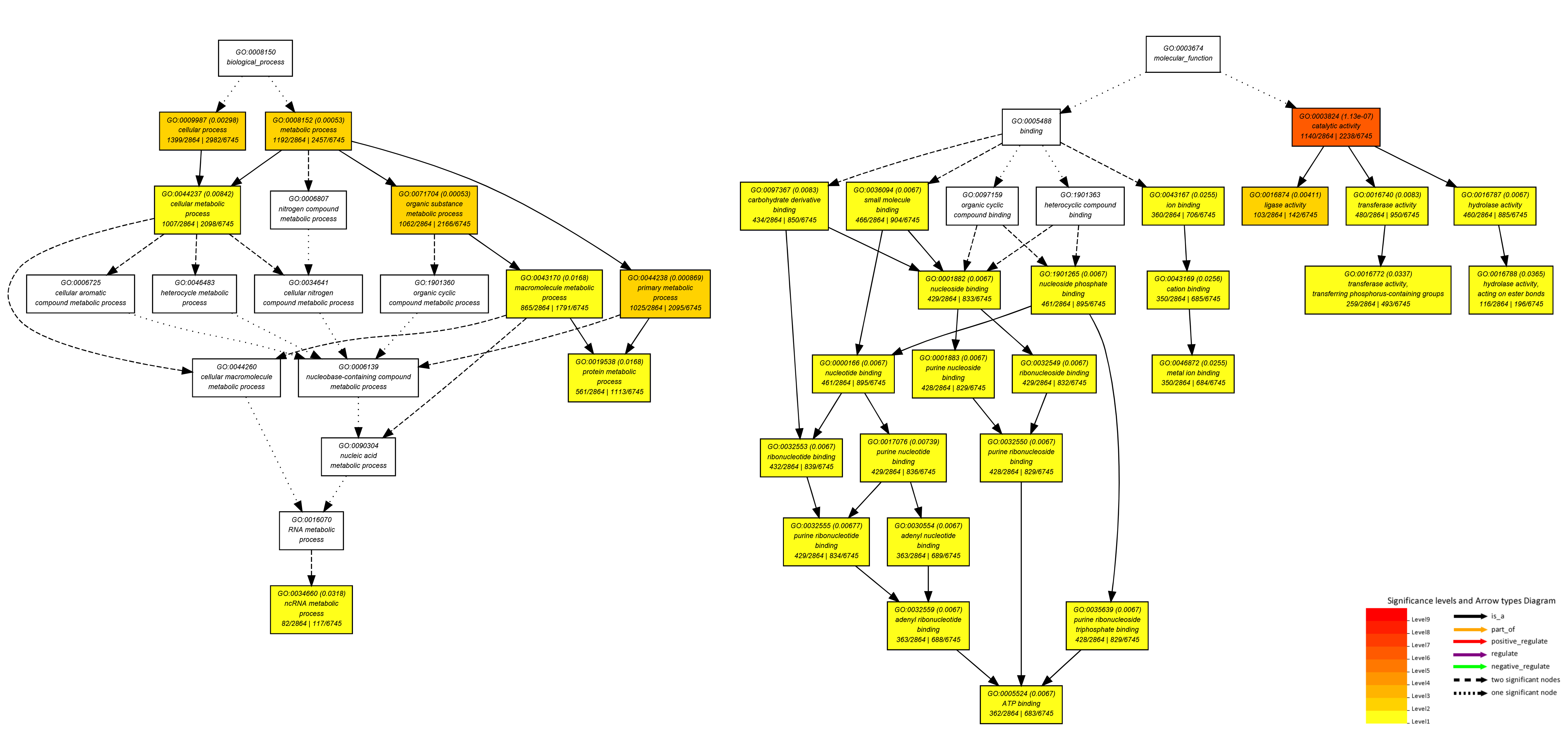

Supplement: Supplementary file 6 — Additional file 6: Figure S7. High-resolution diagram of gene ontology analysis using online analysis toolkit, AgriGO [file 13071_2021_5067_MOESM6_ESM.tif]
